# European Starlings (*Sturnus vulgaris*) as Vectors and Reservoirs of Pathogens Affecting Humans and Domestic Livestock

**DOI:** 10.3390/ani11020466

**Published:** 2021-02-10

**Authors:** Paul R. Cabe

**Affiliations:** Department of Biology, Washington and Lee University, Lexington, VA 24450, USA; cabep@wlu.edu

**Keywords:** European starling, *Sturnus vulgaris*, pathogen, *E. coli*, *Campylobacter*, *Enterococcus*, *Salmonella*, influenza, West Nile virus

## Abstract

**Simple Summary:**

European starlings are an abundant, widespread avian species frequently found in close association with human development and agriculture. Do starlings play a role in transmitting disease to humans or domestic livestock? To investigate the importance of European starlings as disease vectors, I reviewed and assessed the available literature, comprising several hundred published papers. Although a wide variety of potential pathogens have been reported in starlings, the strongest evidence suggests that they may be responsible for harboring and dispersing some species of enteric bacteria, with *Escherichia coli* and *Campylobacter jejuni* of perhaps greatest interest, and primarily in the context of dairies, concentrated animal feeding operations, and other intensive livestock agriculture. Although they can carry other pathogens like *Salmonella* and influenza viruses, evidence suggests they are not as important in the ecology of these diseases.

**Abstract:**

European starlings are an abundant, widespread avian species frequently found in close association with human development and agriculture. The ability of starlings to carry and disperse pathogens of humans and domesticated livestock has received considerable attention, including studies of enteric bacteria, viruses, and some fungi. To investigate the importance of European starlings as disease vectors, I reviewed and assessed the available literature, comprising several hundred published papers. Although a wide variety of potential pathogens have been reported in starlings, the strongest evidence suggests that they may be responsible for harboring and dispersing some species of enteric bacteria, with *Escherichia coli* and *Campylobacter jejuni* of perhaps greatest interest, and primarily in the context of dairies, concentrated animal feeding operations, and other intensive livestock agriculture.

## 1. Introduction

Few bird species have equaled the global success of European starlings (Sturnidae: *Sturnus vulgaris*), native to a broad swath of western Europe but introduced widely [1]. Naturalized populations have expanded to inhabit much of North America, with introduced populations in Australia, New Zealand, South Africa [2], and Argentina [3]. Throughout the range, but especially in areas where they have been introduced, starlings are commensal with humans and are found most frequently and reach highest densities in human dominated landscapes, including agricultural, urban, and suburban areas [1]. European starlings are thus of clear interest in animal-human interactions.

Because of their broad geographic range and close association with humans, their biology and natural history is well known [1]. European starlings are secondary cavity nesters, with 4–6 eggs per clutch and typically two clutches per year. They are broadly and opportunistically omnivorous, eating invertebrates, seeds, and fruits, including grains and prepared livestock foods when available. They are highly social at all times of the year. They readily breed in close proximity to other starlings, and frequently forage in flocks outside of the breeding season; flock sizes of tens of thousands up to hundreds of thousands are relatively common [4] and roosting aggregations can include other species [1] and number in the millions. Migration is variable by population and individual, but collectively, millions of individuals move seasonally in their native range and in North America.

Starlings interact with humans in diverse ways. They are responsible for direct damage to crops, particularly fruits like cherries and grapes, but also some grain crops [5]. They consume and degrade feed provided for livestock, sometimes in prodigious amounts [5]. Starlings can also be a hazard to aviation, and bird strikes with aircraft are well documented [6]. Not all interactions are negative, and starlings are included or portrayed in diverse music and literature [7,8].

Perhaps one of the most important interactions of starlings with humans and animals is in their role as vectors of disease. Starlings can contribute to the spread of many viral, bacterial, and fungal diseases of birds and mammals, including humans. Agricultural and landscape practices can exacerbate the spread of these disease. For instance, cattle dairy and concentrated animal feeding operations which provide abundant food can be very attractive to starlings, especially in the winter when starlings are typically found in larger flocks and the diet includes fewer insects.

There is ample literature on European starlings as potential vectors of diseases which affect humans directly or indirectly through livestock; agricultural impacts include economic losses due to disease in livestock or the spread of human pathogens from starlings to humans via an intermediate animal host. This review covers published findings of human or animal pathogens which may have direct or indirect impacts on humans, but excludes microbes which have no clear interactions with human health and commerce. This review also excludes a variety of microbes which may infect starlings and humans but for which starlings play no clear role in transmission (e.g., *Aspergillus*) to humans or livestock.

## 2. Materials and Methods

The published literature was searched using appropriate search terms (“starling” or “*Sturnus*” plus a variety of general and specific terms dealing with bacterial, viral, and fungal infection) using Scopus (www.scopus.com) and PubMed (pubmed.ncbi.nlm.nih.gov). This literature (250–300 published papers) was sorted and analyzed for relevance to the specific goals of this review.

## 3. Results

Many published studies examined bacterial, viral, and fungal infections in European starlings. A few dealt specifically with direct interactions between starlings and humans, but many of these studies included interactions with livestock and agriculture more generally (Table 1) or were not directly relevant to human-starling interactions.

### 3.1. European Starling and Bacteria Interactions

The vast majority of work on bacterial commensals and pathogens in starlings dealt with species of enteric bacteria which colonize a broad range of vertebrate hosts. Many of these studies included intermediate interactions with domestic livestock including cattle and other ruminants, pigs, and a variety of poultry.

#### 3.1.1. *Escherichia coli*

*Escherichia coli* comprises a large group of related bacteria which colonize the intestinal tract of animals, but may also persist in some external environments. For human–starling interactions, two major concerns exist: the transmission of pathogenic strains directly or indirectly to humans, and the economic losses associated with *E. coli* pathogens spread to livestock. An additional, more general concern is the propagation and spread of antimicrobial resistance genotypes; antibiotic resistant strains of pathogenic bacteria are a serious public health issue, and livestock are often exposed to a variety of antimicrobials [9,10]. 

Many strains are benign, but a number are known to produce mild to severe disease in humans [11]. In cattle, most strains are non-pathogenic but some cause economically important disease [12]; cows also carry strains pathogenic to humans. In pigs, some strains cause economically important disease [13]. To the extent that starlings can spread these bacteria among livestock, pets, or directly to humans, an interest in their role in disease dynamics is warranted. Most research to date has focused on the incidence of pathogenic or antimicrobial resistant strains within wild starling populations and the dynamics of disease spread from starlings to livestock.

Surveys of wild starlings frequently reveal a variety of *E. coli* strains, including pathogenic ones [14]. Up to about 50% of starlings may carry this bacterium, with perhaps nearly a quarter of all individuals carrying pathogenic strains (Table 2) Starlings are clearly able to carry and shed pathogenic *E. coli* strains such as O157:H7 [15]. Circumstantial evidence suggests that they play a role in infecting cattle [16], and molecular typing of strains provides strong evidence of transmission of O157:H7 between starlings and cattle [17,18]. In particular, night roosting aggregations can increase the incidence of this strain in nearby farms [19]. The most likely mode of transmission is contamination of livestock feed and water from starling feces [20,21]. 

Starlings also carry a variety of antibiotic resistant strains [9,22,23,24,25,26,27], at frequencies of 10–20% of individuals (Table 2). Circumstantial evidence suggests that they play a role in spreading these strains to cattle [23,24,25]. Strains resistant to tetracycline, ampicillin, and streptomycin [9], in addition to the fluoroquinolone ciprafloxacin [22,24,25,26] and the cephalosporin cefotaxime [24,25,26] are well documented, and many of these same strains also show resistance to a wide range of other antibiotics, including tetracycline and β-lactam antibiotics [24]. The genetic mechanisms providing resistance are numerous (more than 90 different genes) and are spread over many mechanisms of action, including drug efflux, cellular target alteration, and drug inactivation [9,24]. In at least some cases, plasmids may house the specific resistance genes [9]. In the closely related spotless starling (*Sturnus unicolor*), genes responsible for antibiotic resistance were carried in integrons containing dihydrofolate reductase and aminoglycoside adenyltransferase [27].

#### 3.1.2. *Campylobacter*

*Campylobacter jejuni* is a common intestinal bacteria in birds and mammals, and is a major cause of food poisoning in humans, primarily through undercooked poultry products and unprocessed dairy products. Wild birds are thought to be primary reservoirs of this bacterium [38,39], and starlings have been implicated in its spread [30,40]. Strains of this species may be common among starlings [30,41,42,43], with estimates of incidence as high as 33%, although other estimates are very low (Table 2). Many strains show a strong host association, but the same strains have been identified in starlings and poultry [42,44], starlings and cattle [30,43], and starlings and humans [43], though one study found little evidence that starlings are a major source of infection for domestic poultry [45]. Some strains also cause economically important disease in livestock, including sheep [40]. As with *E. coli*, strains resistant to multiple antimicrobials including ciprofloxacin, erythromycin, and gentamicin are well established in wild starling populations [40], although little is known of the genetic basis for resistance. Although less well studied, direct transmission from feces in urban areas is also plausible [41].

#### 3.1.3. *Salmonella*

*Salmonella* is a genus of enteric bacteria with many serotypes. It is responsible for disease in humans and many mammalian and avian livestock species, including cows, pigs, goats, sheep, and poultry of all types. Concerns about starlings focus mainly on the spread of *Salmonella* among livestock and poultry, though direct transmission to humans via fecal matter in human food crops [28] or urban areas is also a potential concern [46]. For livestock, the concern is both economic loss due to animal illness, and secondarily, transmission to humans via contaminated products (meat, eggs, dairy products, etc.).

Starlings, which are highly vagile, are a potential vector of site-to-site transmission of *Salmonella* [41]. This bacterium is widely reported from starling feces [31,32], which could then contaminate both food and water sources. Starlings may also carry this pathogen externally [47]. Detailed strain identification has demonstrated that specific strains found in cattle at concentrated animal feeding operations can also be found in the wild starlings which visit them, and that the specific strains found did not change over years, implicating starlings as a potential and plausible source of this ongoing contamination [31]. There is some evidence to suggest that winter months have higher potential for disease spread [21], perhaps because of higher densities of starlings at farms and concentrated animal feeding operations. A study in Texas, USA, demonstrated a positive correlation between starling density and *Salmonella* contamination [48].

A large variety of strains have been cultured or identified from European starlings. A study in Australia [13] identified 5 different strains from 6 individuals (*S. enterica enterica* serovar Kottbus, *S. enterica enterica* serovar Muenster, *S. enterica enterica* serovar Bredeney, *S. enterica enterica* serovar Anatum, *S. enterica enterica* serovar Oranienburg.) Carlson et al. [31] identified 5 different strains from 100 individuals, and all strains were recovered both from gastrointestinal cultures and cultures made from external washes. In order of decreasing abundance, these included *S. enterica* Anatum, *S. enterica* Montevideo, *S. enterica* Muenchen, *S. enterica* Kentucky, and *S. enterica* Meleagridis. Gaukler et al. [14] reported a very low incidence of *Salmonella* in free-living starlings, including two isolates *S. choleraesuis* ssp. Arizonae (commonly associated with pigs) and one not serotyped.

Despite compelling evidence that starlings can carry and potentially transmit *Salmonella*, the impact of starlings on livestock disease may be small. The percentage of starlings which test positive for *Salmonella* is often quite low (Table 2), and many details of a compelling argument are missing in many studies [46]. A detailed analysis in Australia found the probability of exposure of domestic pigs to *Salmonella* from starlings to be very low [49]. Clearly, the magnitude of this problem is associated with intensive farming and agricultural practices, and represents an additional potential environmental cost to these practices.

#### 3.1.4. *Enterococcus*

The genus *Enterococcus* comprise a number of species which are typically commensal in intestinal tracts of vertebrates. Some species are opportunistic pathogens of both humans and some livestock. Wild European Starlings frequenting concentrated animal feeding operations harbor many strains; a study [50] of approximately 1400 starlings taken from feedlots across the central United States yielded 658 *Enterococcus* sp. strains, including (in decreasing order of abundance) *E. faecium, E. hirae, E. faecalis, E. casseliflavus, E. gallinarium, E. durans, E. mundtii* and *E. villorum*. These included a large proportion of antibiotic resistant strains: 99% were resistant to erythromycin, 85% to tetracycline, 68% to quinupristin–dalfopristin, 65% to rifampin, 60% to doxycycline, 48% to nitrofurantoin, 16% to fosfomycin, 7% to chloramphenicol, and 1% to ampicillin. Most strains (85–98% of each species) were resistant to multiple drugs; with all strains resistant to 4.3 antibiotic classes on average. Clearly, wild starlings harbor a tremendous array of antibiotic resistant *Enteroccus* strains but their role in disseminating these strains to and among livestock has been little studied and remains poorly understood. There is little published data from which incidence in wild starlings can be estimated.

#### 3.1.5. *Erysipelothrix*

*Erysipelothrix* bacteria can cause disease in some poultry, particularly turkeys but also chickens and waterfowl; pigs may also be affected. They can also cause limited disease in humans. Data is very limited, but this species has been identified in starlings [51]. Their role in disseminating this bacterium to livestock is unknown and likely minimal.

#### 3.1.6. *Mycoplasma*

These bacteria, a group with no external cell wall and very small cell size, include a number of species of potential interest to humans, including pathogens of humans and livestock. One study demonstrated that starlings can carry species potentially detrimental to poultry [52], although another [53] failed to find evidence of infection in starlings. Little is known about their importance in the natural dynamics of these species in wild and captive animal populations.

#### 3.1.7. Tick-Born Human Diseases

Humans are susceptible to a variety of bacterial infections from tick bites; wild vertebrates serve as reservoirs for this pathogens. Migratory passerines, such as European starlings, may be an important part of the overall ecology of these diseases [54]. Some of the tick species utilize birds as hosts, and the European starling’s behavior of extensive foraging on the ground, especially in pastures and fields, suggest potential interactions. There are a very few studies which include starlings. In a western North American study, starlings were negative for *Borrelia*, the pathogen responsible for Lyme disease, but a small percentage were infected with *Anaplasma phagocytophilum* [55], as was the case in Poland [56]. 

### 3.2. European Starling and Viral Interactions

European starlings, like most vertebrates, are hosts to a large and diverse collection of viruses. In the context of interactions with humans and domestic livestock, few generalizations can be made. These pathogens fall under two broad categories, those transmitted directly between individuals and those transmitted among vertebrate hosts by mosquitoes. For these latter, mosquito behavior is a critical component, but outside the scope of this review. It is worth pointing out that these depend on mosquito species which readily or preferably use avian hosts, but also feed on mammalian hosts.

#### 3.2.1. Avian Influenza

Broadly considered, influenza is a tremendously important global disease, with large numbers of cases and substantial mortality in human populations in addition to disease in poultry and pigs. Avian influenza (family *Orthomyxovirdae*, genus *Influenzavirus* A) is endemic in birds, with waterfowl serving as some of the most important reservoir species. In birds, most strains have low pathogenicity, but high pathogenicity strains can impact chickens and other gallinaceous poultry [57]. Humans in close contact with birds may contract avian influenza, often with high mortality rate. Influenza pandemics in the human population may have been directly introduced from birds (as was possibly the case with the 1918 pandemic), or arose through reassortment of avian influenza and strains present in the human population [57]. Many studies have examined prevalence in free-living starlings, which is typically low even in areas with acute outbreaks [37] (see Table 2); Shriner [36] provides a recent review. Starlings are not thought to play a major role in transmission among wild birds [58,59].

#### 3.2.2. Newcastle Disease Virus

Newcastle disease virus (family *Paramyxoviridae*, genus *Orthavulavirus*) causes an eponymous, economically important disease of poultry. It is highly contagious and spreads through direct contact between healthy birds and infected birds or their feces. Starlings have received little attention as possible vectors of this disease, though occasional reports of infection suggest that this would be plausible [60]. Starlings may be infected from domestic poultry and not vice versa [61].

#### 3.2.3. Foot and Mouth Disease Virus (Family *Picornaviridae*, genus *Aphthovirus*)

Foot and mouth disease is a viral disease of commercial importance to livestock farming, though disease in humans is very rare. Primary means of transmission may include close contact, and virus particles on dead tissues or inanimate objects. Starlings have been experimentally infected, implying that birds might play a role in transmission [62], though the evidence is limited and weak.

#### 3.2.4. Avian Pneumovirus (Family *Paramyxoviridae*, Genus *Metapneumovirus*)

Additionally known as avian metapneumovirus, this disease is economically important in poultry, particularly turkeys. Wild birds are thought to be the natural reservoirs, and limited evidence suggests starlings can become infected with this virus [63], though their importance in disease transmission is unknown.

#### 3.2.5. Transmissable Gastroenteritis Virus (Family *Coronavirinae*, Genus *Alphacoronavirus*)

This virus causes an economically important disease in pigs, characterized primarily by diarrhea. Mortality can be very high, and disease severity is typically highest in the youngest individuals. Pilchard [64] demonstrated that artificially infected starlings shed the virus in their feces in amounts capable of producing disease in pigs for up to 32 h. Field studies in England which tracked starlings across pig farms implied that the behavior of free-living starlings could enable the spread of the disease from farm to farm [65].

#### 3.2.6. West Nile Virus

West Nile virus (family *Flaviviridae*, genus *Flavivirus*) is primarily an avian virus that may also infect mammals; it is transmitted via mosquitoes. Serious disease in humans is uncommon but well documented, and infection can be very serious in unvaccinated horses. European starlings can be infected with West Nile Virus, though their importance in spreading the disease to humans is likely limited due to low competence as a reservoir species and feeding behavior of the mosquito vectors [66,67]. Few published studies of surveys with large sample size are available; sporadic reports suggest low seropositivity in wild starlings [13,66,67,68,69] 

#### 3.2.7. Eastern Equine Encephalitis Virus

This virus (family *Togaviridae*, genus *Alphavirus*) is native to the Americas, and is maintained in a variety of avian hosts and spread within birds and to other vertebrates by mosquitoes. Cases of infection or disease in humans are rare but often serious. The virus is also capable of causing mortality in infected horses (equine vaccines are available). In North America, the non-native starling may serve as a highly competent reservoir, and frequently die from infection [70]. As with other mosquito-borne diseases, the European starling’s habits of frequently roosting in large numbers near or over wetlands may provide easy access for mosquitos.

#### 3.2.8. Japanese Encephalitis Virus

This virus (family *Flaviviridae*, genus *Flavivirus*) is a leading cause of viral encephalitis in Asia, including Japan, China, and most regions east to India. Most human infections are asymptomatic, and vaccines are available. In rare cases, encephalitis ensues with a high mortality rate. Important reservoirs for this virus include pigs and wild birds [71]. Although European starlings are not native to this region, they have been introduced to Australia [1], which has experienced a few cases [72]. Experimental evidence from North America suggests that European starlings could possible serve as reservoirs for this virus [73].

#### 3.2.9. Usutu Virus

This flavivirus (family *Flaviviridae*, genus *Flavivirus*) has rapidly expanded its range outside of Africa, and is currently under scrutiny as a potential break-out pathogen in humans. To date, human infections are exceedingly rare and often asymptomatic [74]. Similar to West Nile and eastern equine encephalitis virus, birds serve as the primary reservoir and mosquitoes are the primary vector. Wild European starlings have been shown to be infected [75], though their importance in the maintenance and spread of this virus is unknown.

### 3.3. Fungal Pathogens

#### Histoplasma

This common soil fungus is widespread but most common in the Americas. It is particularly associated with fecal deposits from birds and bats. Inhalation of spores can cause lung disease in humans. Starling roosts can generate large amounts of fecal material, and have been associated with histoplasmosis in humans [76,77,78,79,80], but the problem is not specific to starlings. Interestingly, all of the published accounts date from the 1960s.

## 4. Discussion

European starlings have been studied to determine their role in disease dynamics for a broad array of bacterial and viral diseases. Their popularity as a research species may result from their frequent association with humans and agriculture and thus their potential importance as vectors of disease. Their popularity as research subjects may also be due to their ease of use—they are frequently numerous, easily located in human-dominated landscapes, and in some areas of introduction, they are excluded from legislation protecting native wild species, making permits and other logistical considerations of avian field research less difficult. Regardless, starlings are associated with human development and agricultural operations across a number of continents, making their potential roles in the spread of disease very important.

The single largest impact is likely to be from the spread of pathogenic enteric bacteria from wild reservoirs to livestock and vice versa. Enteric illnesses are the second most abundant category of communicable human diseases, and much concern has been focused on the importance of wild birds as reservoirs and vectors of these pathogens [46]. In addition to the direct human health burden, these diseases account for a tremendous cost to the livestock industry; the total cost of livestock diseases associated with alien species in the USA was estimated in the tens of billions of dollars in 2005 [81], although starlings are directly responsible for only a small fraction of this. 

The evidence for the starling’s importance in the spread and dynamics of bacterial pathogens is perhaps strongest for *E. coli* and *C. jejuni*. While strong evidence links European Starlings to *Salmonella*, their overall importance in the transmission of this pathogen to humans and livestock may be limited [46,50]. 

European Starlings may also play a role in the persistence and transmission of a number of viral diseases in both their native and introduced ranges, though in general, fewer studies have examined this topic. Influenza has received more attention, no doubt due to the high health burden of influenza in the human population. In some of the other more important examples, the viruses are spread through mosquitoes, and depend on mosquitoes with a host range which includes both mammals and birds. In all these cases, the impact of starlings relative to other avian species is often not clearly known, and there is no evidence that starlings are uniquely important.

Clearly, the available literature reflects a preoccupation with interactions between European starlings and intensive livestock agriculture. In this light, starlings are just one additional concern about practices that have manifold impacts on human health [82] and a tremendous impact on the environment [83,84]. Very little is known about their potential impacts on less intensive animal agriculture, though limited evidence suggests that small scale livestock production on diversified farms may reduce the prevalence of pathogens due to starlings and other introduced bird species [28].

## 5. Conclusions

European starlings have a broad global range due to human introductions. Adaptable and resilient, they thrive in close association with human agriculture and development and are an abundant species in many cities, suburbs, and agricultural areas. Their potential for spreading pathogens directly or indirectly to humans is clearly of interest, as is the economic damage caused by the dispersing of diseases to livestock. A number of studies have confirmed that starlings can carry bacterial enteric pathogens of both humans and domestic livestock. Circumstantial evidence, for example the presence of identical strains in livestock and free-living starlings, suggests they may be important vectors for these diseases. Much of the research to date has targeted intensive animal agriculture, including concentrated animal feeding operations and large dairies, but little is known about their effects on small-scale animal agriculture. In this context, European starlings and pathogen dispersal can be added to the long litany of health, environmental, and ethical concerns associated with intensive animal agriculture. 

## Figures and Tables

**Table 1:** Publications which include European starlings and bacterial, viral, and fungal diseases which impact humans and domestic livestock and are available from two scientific databases (entries of 0 in the first two columns indicate that these specific search terms yielded no results; a few publications were located through more general searches).

Search Terms in Addition to “European Starling” or *Sturnus*	Pubmed	Scopus	Including Domestic Livestock/Agriculture (in Scopus)
*E. coli*	20	22	16
*Salmonella*	17	23	14
*Campylobacter*	11	15	7
*Enterococc* *	2	4	1
*Erysipelothrix*	0	0	0
*Mycoplasma*	6	6	1
*Borrelia* or *Anaplasma*	1	2	0
influenza	13	17	2
“West Nile virus”	9	14	0
“Equine encephalitis”	2	2	0
Usutu and virus	1	1	0
Newcastle and virus	3	5	1
“Foot and mouth”	1	1	1
Pneumovirus	0	0	0
Histoplasmosis	0	0	0

**Table 2:** Incidence of some pathogens in wild European starlings. Caution is urged in interpreting these data due to the wide variety of research design, methodology, and geography.

Pathogen	Incidence (N)	Notes	Reference
*E. coli*	36.6% (473)	Australia, commercial piggeries	[13]
*E. coli*, all strains Antibiotic resistant strains	35% (26) 19.2%	Ireland	[9]
*E. coli* *E. coli* O157:H7	48.2% (434) 0	central North America, cattle CAFO	[14]
*E. coli*, pathogenic strains	23% (87)	western North America, sampled from vegetable farms	[28]
*E. coli* O157:H7	1.2% (430)	Ohio, USA, dairy farms	[18]
*E. coli*, pathogenic strains	23% (87)	western North America, sampled from vegetable farms	[28]
*E. coli* O157:H7	2.8%	Sampled from pooled feces, North America dairy farm	[21]
*E. coli*, ciprafloxin resistant	10.2% (1477)	cattle CAFO, Texas, USA	[23]
*Campylobacter* sp.	20.7 (87)	western North America, sampled from vegetable farms	[28]
*C. jejuni*	13.2% (174)	Eastern North America, small ruminant farm	[29]
*C. jejuni*	0.6% (473)	Australia, commercial piggeries	[13]
*C. jejuni*	33% (150)	midwestern USA cattle feedlots	[30]
*Salmonella* sp.	0% (87)	western North America, sampled from vegetable farms	[28]
*Salmonella* spp.	1.3% (473)	Australia, commercial piggeries	[13]
*Salmonella* spp.	32% (100)	North America, cattle CAFO	[31]
*Salmonella* sp.	5.9% (34)	Texas, USA	[32]
*Salmonella* sp.	17% (100)	carried externally	[31]
*Salmonella* sp.	1.12% (179)	from pooled feces	[21]
*Salmonella* sp.	0.2% (174)	Eastern North America, small ruminant farm	[29]
*Salmonella enterica*	2.5% (?)	Texas USA, cattle CAFO	[33]
*Salmonella* sp.	0.7% (434)	central North America, cattle CAFO	[14]
*Salmonella* sp.	1%	feces collected beneath a roost, Switzerland	[34]
Avian Influenza virus	0%	Australia, commercial piggeries	[13]
Avian Influenza virus	1.5% (328)	Ohio, USA	[35]
Avian Influenza virus, seropositive virus/RNA detection	0.09% (1173) 0% (864)	compilation of studies across North America	in [36]
Avian Influenza virus, H5	1.4%(69)	Iowa, USA, during poultry farm outbreak	[37]

## Data Availability

Not applicable.
